# Physical activity reduces the risk of pneumonia: systematic review and meta-analysis of 10 prospective studies involving 1,044,492 participants

**DOI:** 10.1007/s11357-021-00491-2

**Published:** 2021-11-25

**Authors:** Setor K. Kunutsor, Samuel Seidu, Jari A. Laukkanen

**Affiliations:** 1 National Institute for Health Research Bristol Biomedical Research Centre, University Hospitals Bristol and Weston NHS Foundation Trust and University of Bristol, Bristol, UK; 2 Musculoskeletal Research Unit, Translational Health Sciences, Bristol Medical School, University of Bristol, Learning & Research Building (Level 1), Southmead Hospital, Bristol, BS10 5NB UK; 3 Department of Medicine, Central Finland Health Care District, Jyväskylä, Finland; 4 Diabetes Research Centre, University of Leicester, Leicester General Hospital, Gwendolen Road, Leicester, LE5 4WP UK; 5 Leicester Diabetes Centre, Leicester General Hospital, Gwendolen Road, Leicester, LE5 4WP UK; 6 Institute of Public Health and Clinical Nutrition, University of Eastern Finland, Kuopio, Finland; 7 Faculty of Sport and Health Sciences, University of Jyväskylä, Jyväskylä, Finland

**Keywords:** Physical activity, Pneumonia, Cohort study, Risk factor, Systematic review, Meta-analysis

## Abstract

The beneficial effects of regular physical activity in promoting health and preventing chronic diseases are well documented. The relationship between regular physical activity and the risk of pneumonia is uncertain. We aimed to evaluate the magnitude and specificity of the prospective association between regular physical activity and the risk of pneumonia using a systematic review and meta-analysis of published observational cohort studies in general populations. Relevant studies with at least 1 year of follow-up were sought from inception until 15 September 2021 in MEDLINE, Embase, Web of Science, and manual search of relevant articles. Relative risks (RRs) with 95% confidence intervals (CIs) for the maximum versus the minimal amount of physical activity groups were pooled using fixed effects meta-analysis. The quality of the evidence was evaluated using the GRADE tool. A total of 10 prospective cohort studies comprising 1,044,492 participants and 7681 events were eligible. The pooled multivariable-adjusted RR (95% CI) of pneumonia comparing the most versus the least physically active groups was 0.69 (0.64–0.74). This association was significantly modified by type of outcome (*p-*value for meta-regression = .002): 0.82 (0.72–0.93) for incident pneumonia and 0.64 (0.59–0.70) for pneumonia-related mortality. There was no evidence of heterogeneity and publication bias. The GRADE quality of the evidence ranged from moderate to low. Aggregate analysis of 10 cohort studies shows that regular physical activity is associated with lowered risk of incident pneumonia and pneumonia-related mortality in the general population. Physical activity types that are attractive to and feasible for high-risk populations need to be identified and encouraged. Systematic review registration: PROSPERO 2021: CRD42021277514.

**Supplementary Information:**

The online version contains supplementary material available at 10.1007/s11357-021-00491-2.

## Introduction

Pneumonia is an inflammatory condition of the lung tissue commonly caused by bacteria or viruses [1]. It can be acquired in the community (community-acquired pneumonia, CAP) or in the hospital environment (hospital acquired pneumonia, HAP) [2]. Pneumonia is a leading cause of mortality among older people, the young, and people with comorbidities [1]; in 2016, pneumonia was the fourth leading cause of death globally, accounting for 3 million deaths [3]. Pneumonia is also associated with considerable morbidity, reduced quality of life, and high healthcare costs [2]. The annual global economic cost of CAP has been estimated to be $17 billion [4]. Major risk factors for pneumonia constitute smoking, excessive alcohol consumption, respiratory conditions such as asthma and chronic obstructive pulmonary disease (COPD), and other chronic conditions such as diabetes, kidney and liver disease [4]. Pneumonia constitutes a substantial public health burden globally and is a preventable cause of death and disability. The beneficial effects of regular physical activity in promoting health and preventing vascular and non-vascular diseases are well documented [5–7]. Regular physical activity also reduces the risk, duration or severity of infectious diseases [8] and has been shown to improve acute and long-term prognosis of pneumonia in older patients [9]. Data on the prospective association between physical activity and risk of pneumonia has been sparse and divergent. Whereas, some studies have reported associations between physical activity and risk of pneumonia [10–12], others have reported no evidence of an association [13–15]. The reasons for the null associations in some of these studies are uncertain but could be related to inadequate power (low event rates), effects of confounding, or could just be a true association. In a systematic review that quantified the association of habitual physical with the risk of community-acquired infectious disease, laboratory-assessed immune parameters, and immune response to vaccination, pooled analysis of 6 studies showed that habitual physical activity was associated with a 31% risk reduction in community-acquired infectious disease, with a 37% risk reduction in infectious disease mortality in pooled analysis of 4 studies [16]. This review, however, focused on a broad range of clinically diagnosed infections and their complications and included a combination of outcomes such as pneumonia, blood stream infections and sepsis. Though habitual physical activity reduced the risk of these infectious disease outcomes, it is uncertain which specific outcome was driving the association. Additionally, no subgroup analysis was conducted for the specific outcome of pneumonia. There is uncertainty as to whether physical activity is associated with the risk of pneumonia. It would be useful to conduct a pooled analysis of available published prospective evidence on the associations which would provide the opportunity to re-evaluate the relationship in a larger representative sample of participants, but this has not yet been undertaken. In this context, we sought to evaluate the magnitude and specificity of the association between physical activity and future risk of pneumonia in the general population (non-athletes) using a systematic review and meta-analysis of all published observational cohort studies to date.

## Materials and methods

### Data sources and searches

**Fig. 1:**
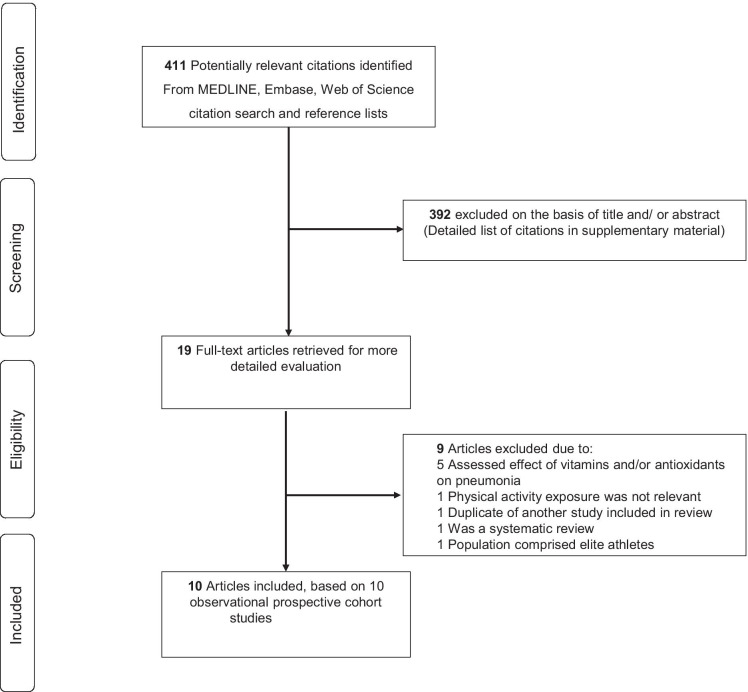
PRISMA flow diagram

The protocol for this this systematic review and meta-analysis was registered in the PROSPERO prospective register of systematic reviews (CRD42021277514). The review was conducted according to PRISMA and MOOSE guidelines [17, 18] (Electronic Supplementary Materials 1–2) (Fig. 1). We searched MEDLINE and Embase (using OvidSP interface) from inception to 15 September 2021 with no restrictions on language. We planned to translate all non-English language studies using translators or the “Google Translate” service. The computer-based searches used a combination of keywords or terms relating to the exposure (“physical activity”, “exercise”, “aerobic training”) and outcome (“pneumonia”, “lower respiratory tract infection”). The full search strategy is presented in Electronic Supplementary Material 3. One author (SKK) initially screened the titles and abstracts of the retrieved references to assess the potential for inclusion in the review. Screening was conducted using Rayyan, a free online bibliographic tool that helps expedite the initial screening of abstracts and titles using a process of semi-automation while incorporating a high level of usability.[19] This was then followed by full-text evaluation of the selected titles and abstracts. This was independently conducted by two authors (SKK and SS), with disagreements resolved in consultation with a third author (JAL). To identify potential articles missed by the search strategy, the reference lists of relevant studies and review articles were manually scanned and citing references were also checked in Web of Science.

### Study selection

We included all observational population-based observational cohort (retrospective or prospective) studies that had evaluated the relationship of physical activity with the risk of pneumonia in adult general populations and had at least 1 year of follow-up. We excluded the following studies: (i) case–control study designs because of lack of temporality, (ii) those involving elite athletes and/or evaluated competitive or endurance sports, and (iii) those evaluating the associations between measures of fitness (e.g., cardiorespiratory fitness, physical fitness, exercise capacity) and risk of pneumonia.

### Data extraction and risk of bias assessment

Using a standardised data collection form, the lead author (SKK) initially extracted relevant data from eligible studies and a second author (SS) independently checked the data using the original articles. A third author (JAL) was involved to help resolve any disagreements. Data on the following study characteristics were extracted: first author surname and year of publication, geographical location, year of enrolment/data collection, study design, demographic characteristics (age and percentage of males), sample size, duration of follow-up, assessment of physical activity, risk comparisons, number of outcome events, the most fully adjusted risk ratios of outcomes (and corresponding 95% confidence interval [CIs]), and list of covariates adjusted for. The level of adjustment was defined as ‘ + ’ minimally adjusted analysis, i.e. age and/or sex; ‘ +  + ’ as adjustment for established risk factors without inflammation, i.e. age and/or sex plus body mass index (BMI), socioeconomic status, alcohol consumption, smoking, and comorbidities; and ‘ +  +  + ” as adjustment for established risk factors including inflammation. We also extracted data on minimally adjusted estimates (unadjusted, age, or age and sex) to be used for sensitivity analysis. When there were multiple publications of studies using data of the same cohorts, the study selection was based on a single set of most comprehensive results to avoid double counting of a cohort in the pooled analysis. The most up-to-date comprehensive study was used (i.e. the one with the most extended follow-up or analysis covering the largest number of participants and events). The Cochrane Risk of Bias in Non-randomised Studies–of Interventions (ROBINS-I) tool was used to assess the risk of bias within individual observational studies [20]. This tool assesses risk of bias for the following domains: confounding, participant selection, classification of interventions, deviations from intended interventions, missing data, outcome measurements, and selective reporting. The risk is quantified in each domain as low risk, moderate risk, serious risk, or critical risk; then, an overall judgement of the risk of bias is provided for each study. Finally, to grade the quality of evidence across outcomes, we used the Grading of Recommendations Assessment, Development and Evaluation (GRADE) tool, a widely adopted reproducible and transparent framework for grading certainty in evidence and used in clinical decision making [21]. GRADE considers the following criteria: study limitations, inconsistency of effect, imprecision, indirectness, and publication bias, and has four levels of evidence: very low, low, moderate, and high.

### Data synthesis and analysis

Summary measures of association were reported as relative risks (RRs) with 95% CIs. All studies categorised physical activity exposure (e.g., leisure-time physical activity, total or any physical activity) into two or more groups. There was varied reporting of risk comparisons; hence, the need to provide some consistency to enhance interpretation of the findings. Since the risk estimates could not be transformed into consistent comparisons, the extreme groups (i.e., top versus bottom or maximum versus the minimal amount of physical activity) reported for each study were used for the analyses. This approach, which we have utilised in previous similar meta-analyses [22–25], is considered reliable as it has been shown that pooled estimates from transformed and untransformed data are qualitatively similar [26]. When a study assessed specific types of physical activity in addition to total or any physical activity, we only used risk estimates for total or any physical activity in the pooled analysis. Standard chi-square tests and the *I*^2^ statistic were used to quantify the extent of statistical heterogeneity across studies [27, 28]. Given the absence of substantial heterogeneity among contributing studies, RRs were pooled using a fixed effects model. We explored for the effect of interactions on the association using study-level characteristics such as geographical location (Europe vs. North America vs. Asia), sex (men vs. women), the average age at baseline (≥ 55 vs. < 55 years), the average duration of follow-up (< 10 vs. ≥ 10 years) based on the distribution of the data, type of pneumonia outcome (incident pneumonia vs. pneumonia-related mortality), number of events (≥ 350 vs. < 350), and degree of adjustment (+ vs. + +), which was conducted using stratified analysis and random effects meta-regression [29]. To test the robustness of the observed association, we conducted a sensitivity analysis by investigating the influence of omitting each study in turn on the overall result (stata module metaninf). As a sensitivity analysis, we also pooled studies that reported minimally adjusted estimates (age and/or sex adjusted). To explore for small study effects, we visually inspected constructed Begg’s funnel plots [30] and performed Egger’s regression symmetry test [31]. We employed Stata version MP 17 (Stata Corp, College Station, Texas) for all statistical analyses.

## Results

### Study identification and selection

The search of databases, manual screening of relevant articles and Web of Science citation search identified 411 potentially relevant citations. Following initial screening of titles and abstracts, 392 citations were excluded (Supplementary Material), which left 19 articles for full-text evaluation. After full-text evaluation, 9 articles were excluded because (1) exposure was not relevant (*n* = 6), (2) study was a review (*n* = 1), (3) study was based on a cohort already included in the review (*n* = 1), and (4) population was based on elite runners and walkers (*n* = 1). Full details of reasons for exclusions during full-text evaluation are provided in Electronic Supplementary Material 4. Overall, 10 articles based on 10 studies were included in the review [10, 11, 13–15, 32–36]. The included studies comprised 1,044,492 participants and 7681 events. All relevant studies identified during the review were reported in the English language; hence, no translations were required.

### Study characteristics

Table 1 summarises the study design and population characteristics of the eligible studies evaluating the associations between physical activity and the risk of pneumonia. All the studies were based on prospective cohort study designs. Though there was no restriction on publication date, publication years of eligible studies ranged from 2000 to 2021. Six studies reported on incident pneumonia and four on pneumonia-related deaths. For studies that reported data on average age of participants at baseline, these ranged from approximately 36 to 69 years, with a weighted mean of 55.8 years. Seven studies enrolled both men and women, 2 studies only men, and 1 study only women. Four studies were based in Europe (Finland, Norway and the UK), 4 in North America (USA), and 2 in Asia (Japan). The average duration of follow-up ranged from 2.0 to 14.8 years, with a weighted mean of 9.7 years. All 10 studies assessed physical activity through self-reported questionnaires, but the categorisation of physical activity varied across studies. Most studies reported physical activity as a combination of aerobic and resistance activities. A detailed description of ascertainment of physical activity exposures by individual studies is reported in Electronic Supplementary Material 5. The degree of confounder adjustment varied across studies, but all studies except for one, adjusted for established risk factors such as age, sex, BMI, socioeconomic status, alcohol consumption, smoking, and comorbidities. None of the studies adjusted for inflammation.

**Table 1:** Baseline characteristics of observational cohort studies included in review

Author, year of publication	Study name	Country	Year of study	Male %	Mean age, yr	Follow-up, yr	PA exposure, type	Risk comparisons	No. of cases	No. of participants	Endpoint	Complete adjustment
Hamer, 2019	HSE/SHS	UK	1994–1995/1997–1999/2003–2004/2006/2008	46.6	47.1	9.4	Total PA, Aerobic plus resistance	Sufficiently active vs inactive	579	97,844	Pneumonia deaths	Age, sex, longstanding illness, social occupational status, and mutually for physical activity, BMI category, cigarette smoking, alcohol
Inoue, 2007	JACC Study	Japan	1988–1990	41.9	57.8	10.0	Playing sports, Aerobic plus resistance	> 4 vs 1–2 h/week	1246	110,792	Pneumonia deaths	Age and history of DM
Neuman, 2010	NHS II	USA	1989–1989	0	36.0	12.0	Total PA, Aerobic plus resistance	Top vs bottom quintile	1265	83,165	Incident pneumonia	Age, BMI, smoking, and alcohol use
Baik, 2000	HPFS	USA	1990	100.0	56.4	6.0	Total PA, Aerobic plus resistance	Top vs bottom quintile	290	26,429	Incident pneumonia	Age, smoking, and alcohol intake
Paulsen, 2017	HUNT2	Norway	1995–1997	46.8	48.6	14.8	Total PA, Aerobic plus resistance	High vs none	186	64,027	Incident pneumonia	Age and sex
Hemila, 2006	ATBC	Finland	1985–1993	100	NR	3.0	Leisure PA, Aerobic plus resistance	Heavy vs light	65	7835	Incident pneumonia	Age, BMI, smoking status, duration of smoking, coffee, and alcohol consumption
Ogunmoroti, 2016	MESA	USA	2000–2002	47.2	62.0	10.2	Total PA, Aerobic plus resistance	Ideal vs poor	334	6506	Incident pneumonia	Age, sex, race/ethnicity, education, and income
Ahmadi, 2021	UK Biobank	UK	2006–2010	45.4	56.5	11.3	Total PA, Aerobic plus resistance	Sufficient vs inactive	3170	468,569	Pneumonia deaths	Age, sex, socioeconomic status, ethnicity, BMI, corticosteroid use, and comorbidities (CVDs, cancers, diabetes, chronic respiratory disease, liver disease, end-stage renal disease, immune disorders/HIV, and hypertension)
Ikeda, 2020	Japanese Specific Health Checkup	Japan	2008	39.2	69.2	3.4	Walking, Aerobic	Walking vs no walking habits	145	177,075	Pneumonia deaths	Age, sex, BMI, smoking status, alcohol drinking habits, past history of heart diseases and stroke, hypertension, DM and residential municipalities
Jackson, 2016	ACT	USA	1994–1996/2000–2003	40.0	NR	2.0	Exercise, Aerobic	4–7 vs 0 days/week	401	2250	Incident pneumonia	Age, sex, COPD, CHF, use of home oxygen, difficulties in dressing or walking half a mile, history of alcohol-related aggressive behavior, BMI, and use of corticosteroids

*BMI*, body mass index; *CAP*, community-acquired pneumonia; *CHF*, congestive heart failure; *COPD*, chronic obstructive pulmonary disease; *CVD*, cardiovascular disease; *DM*, diabetes mellitus; *HIV*, human immunodeficiency virus; *PA*, physical activity

Study abbreviations: *ACT*, Adult Changes in Thought; *ATBC*, Alpha-Tocopherol, Beta-Carotene Cancer Prevention; *HPFS*, Health Professionals Follow-up Study; *HSE/SHS*, Health Survey for England/Scottish Health Survey; *HUNT*, Nord-Trøndelag Health Study; *JACC*, Japan Collaborative Cohort Study for Evaluation of Cancer Risk; *MESA*, Multi-Ethnic Study of Atherosclerosis; *NHS*, Nurses Health Study

### Risk of bias of included studies using ROBINS-I tool

One study was at moderate risk of bias (i.e. at low or moderate risk of bias for all domains) and 9 studies were at serious risk of bias (i.e. were judged to be at serious risk of bias in at least one domain, but not at critical risk of bias in any domain) (Electronic Supplementary Material 6).

### Physical activity and risk of pneumonia

**Fig. 2:**
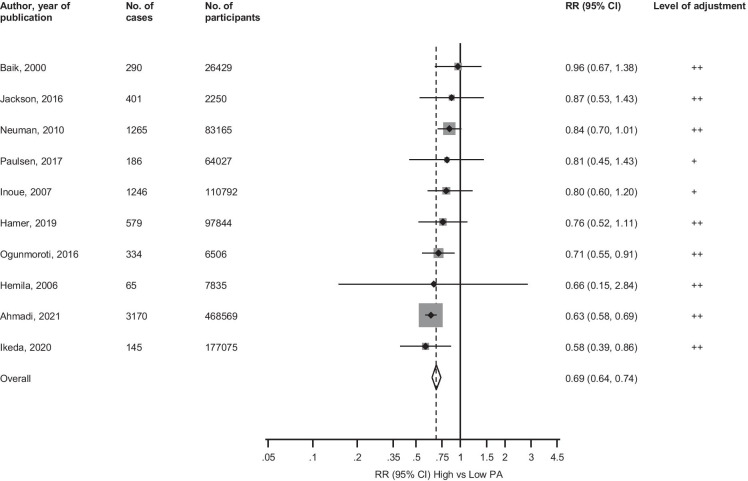
Observational cohort studies of physical activity and risk of pneumonia included in the meta-analysis. The summary estimate presented was calculated using fixed effects models and was based on fully adjusted estimates; sizes of data markers are proportional to the inverse of the variance of the relative ratio; CI, confidence interval (bars); PA, physical activity; RR, relative risk. The level of adjustment was defined as ‘ + ’ minimally adjusted analysis, i.e. age and/or sex; ‘ +  + ’ as adjustment for established risk factors without inflammation, i.e. age and/or sex plus body mass index, socioeconomic status, alcohol consumption, smoking, and comorbidities

**Fig. 3:**
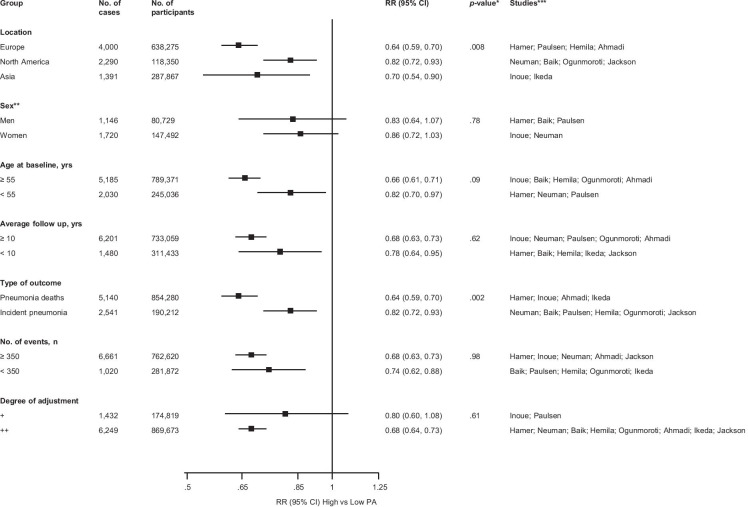
Relative risks for pneumonia comparing maximal versus minimal amount of physical activity, grouped according to several study-level characteristics The summary estimates presented were calculated using fixed effects models; CI, confidence interval (bars); PA, physical activity; RR, relative risk; a single asterisk (*), *p*-value for meta-regression; double asterisks (**), analysis is based on 3 studies in men and 2 studies in women; triple asterisks (***), studies included in each subgroup

The pooled multivariable-adjusted RR (95% CI) of pneumonia comparing the most physically active versus the least physically active groups was 0.69 (0.64–0.74) (Fig. 2). There was no evidence of substantial heterogeneity between the contributing studies (*I*^*2*^ = 39%, 0 to 71%; *p* = 0.09). In subgroup analysis, the strength of the associations did not appear to differ according to several study-level characteristics, except for evidence of effect modification by location (*p*-value for meta-regression = 0.008) and type of outcome (*p*-value for meta-regression = 0.002); the associations seemed to be stronger for studies conducted in Europe and those that evaluated pneumonia-related mortality (Fig. 3). Exclusion of any single study at a time from the meta-analysis did not change the direction of the association, yielding pooled RRs (95% CIs) which ranged from 0.66 (0.62–0.72) to 0.79 (0.71–0.88) (Electronic Supplementary Material 7). Five studies reported age and/or sex adjusted estimates; the RR (95% CI) for pneumonia in their pooled analysis was 0.68 (0.63–0.74) (Electronic Supplementary Material 8). Due to the possibility of some participants overlapping in 2 studies conducted in the UK (one based on the Health Survey for England/Scottish Health Survey (HSE/SHS) [13] and the other on UK Biobank [32]), we conducted specific sensitivity analyses. On exclusion of the HSE/SHS, the RR (95% CI) for pneumonia was 0.68 (0.64–0.73). When the UK Biobank study (which was also the biggest study) was excluded, the RR (95% CI) was 0.79 (0.71–0.88).

### Publication bias

A funnel plot of the 10 studies reporting on the associations between physical activity and risk of pneumonia showed no evidence of asymmetry (Electronic Supplementary Material 9), which was consistent with Egger’s regression symmetry test (*p* = 0.09). There was no evidence of such selective reporting when studies were grouped by size in meta-regression analysis (Fig. 3).

### GRADE summary of findings

GRADE ratings for the overall population and for pneumonia incidence and pneumonia-related mortality were assessed and are reported in Electronic Supplementary Material 10. GRADE quality of the evidence ranged from moderate to low.

## Discussion

### Summary of main findings

In a pooled analysis of 10 population-based prospective cohort studies comprising over one million participants, we found strong and convincing evidence of an association between regular physical activity and lowered risk of pneumonia. The association was robust and consistent in several sensitivity analyses including exclusion of the single largest study [32] and across several relevant subgroups including incident pneumonia and pneumonia-related mortality, though the association appeared to be stronger for the latter. The quality of the evidence ranged from moderate to low.

### Comparison with previous work

A detailed literature search did not identify any previous meta-analysis that had aggregated the existing data on the relationship between physical activity and the specific outcome of pneumonia. Hence, it is difficult to make any head-to-head comparisons in the context of a previous review. However, in a meta-analysis that was just published this year, Chastin and colleagues evaluated the association of habitual physical with the risk of community-acquired infectious disease, laboratory-assessed immune parameters, and immune response to vaccination [16]. In addition to not evaluating the specific outcome of pneumonia, only 6 studies were included in their pooled analysis. Our findings represent new data on the relationship between physical activity and pneumonia and are based on the most up-to-date evidence in general population participants and limited to prospective cohort study designs.

### Possible explanations for findings

Regular physical activity has been well documented to reduce the risk of several chronic diseases as well as mortality [5, 6]. The pathophysiological mechanisms underlying these associations may relate to the ability of physical activity to (i) improve levels of potential risk factors such as body weight, hypertension, lipids, haemostatic factors, adipokines, and sex hormones [37, 38] and (ii) decrease systemic inflammation [39, 40]. There is also mounting epidemiological evidence on the relationship between regular physical activity and a reduction in the incidence, duration, or severity of infections [8]. The potential mechanisms of action underlying the protective effect of physical activity on infections include stimulation of the antipathogen activity of immune system macrophages and key immune system cells in the blood as well as suppressing inflammation in the lungs [8]. Even free-living daily physical activities such as walking have been shown to enhance immune function in older populations [41, 42]. Pneumonia is a respiratory tract infection and characterized by inflammation [43]; hence, all these mechanisms may underpin the observed relationships between regular physical activity and reduced risk of incident pneumonia and pneumonia-related mortality. Physical activity also has direct effects on target organs and tissues [44]; the increasing demand of ventilation during progressive physical exercise may mechanically improve and increase the amount of ventilation in pulmonary airways, bronchioles and alveoli, which subsequently improves pulmonary function.

### Implications of findings

These findings have important clinical implications. They add to the overwhelming evidence on the benefits of regular physical activity on chronic diseases, infectious diseases, and mortality due to these diseases; physical activity has substantial benefits on overall health with the ability to subsequently reduce healthcare expenditures [45]. Though physical activity guidelines recommend 150–300 min/week of moderate-intensity or 75–150 min/week of vigorous-intensity aerobic physical activity/ exercise for adults, as these levels are associated with substantial benefits in the majority of people [46], most populations do not achieve these levels. It has been reported that in the USA, only 46% of adults meet the general physical activity recommendations [47]. A systematic review which evaluated the sedentary behavior of older people found out that approximately 60% of older adult’s reported sitting for more than 4 h per day [48]. Pneumonia is a leading cause for hospitalisation and subsequent mortality among older people [49]; older people with sedentary behavior are more at risk for pneumonia and its related outcomes. Hence, there is a need to identify physical activity types or sports that are attractive to and feasible for these population groups to ensure regular physical activity. Physical activity in any form has health benefits; even the least active behavior which is standing has been suggested to alleviate the health risks associated with prolonged sitting [50]. Walking is a physical activity which is common among older people and has been shown to have several health benefits, including reducing the risk of pneumonia-related mortality [34]. This activity should be promoted extensively in clinical practice and via population wide approaches.

### Strengths and limitations

Several strengths of this systematic review include the (i) novelty; (ii) adoption of a comprehensive search of all the major databases and manual screening of relevant articles without restrictions on the language or year of study, which minimised the likelihood of missing any relevant study conducted on the topic; (iii) inclusion of observational prospective cohort studies, which are characterised by temporality; (iv) exploration for evidence of effect modification using clinically relevant characteristics, evaluation for small study effects, and several sensitivity analyses; (v) no evidence of substantial heterogeneity and publication bias among contributing studies; and (vi) assessment of the risk of bias and the quality of the evidence using well-established tools. Some important limitations deserve consideration, even though most were inherent to the included studies. First, though systematic review guidelines suggest the use of at least two investigators in initial screening of abstracts and titles as this may reduce the possibility of rejecting relevant reports,[51], we adopted a pragmatic approach of using one experienced reviewer for the initial screening due to manpower constraints. To ensure relevant records were not missed, an experienced reviewer was involved in the initial screening and a broad inclusion approach was used; only citations that did not clearly satisfy the inclusion criteria were excluded. We also employed a well-recognised online tool that helps expedite the screening of abstracts and titles using a process of semi-automation. Second, there was variation in the assessment and categorisation of physical activity exposures by the original studies, which did not enable transformation into consistent comparisons; hence, comparisons could only be made between the most and least active. Nevertheless, we have shown in a previous study that pooled results from untransformed data of extreme categories are not very different from results based on transformed data [26]. Third, whether the association varied by the type of physical activity (aerobic vs. resistance) could not be evaluated because most studies reported physical activity as a combination of the two types. Fourth, the actual dose–response relationship of the association could not be evaluated because of the heterogeneous nature of the physical activity data. Hence, it is uncertain which amount and intensity of physical activity is essential for the prevention of pneumonia. Nevertheless, given that studies involving elite athletes, competitive or endurance sports were not included, these findings can be generalised to general population participants for whom the physical activity recommendations of 150–300 min/week of moderate-intensity or 75–150 min/week of vigorous-intensity aerobic PA/ exercise for adults, apply [46]. It has also been shown that the protective effect of physical activity on mortality related to infectious diseases such as pneumonia, is apparent at low levels of activity (moderate-intensity walking for 30 min/week) far below guideline recommendations [13]. Fifth, physical activity was self-reported and hence the potential for misclassification bias, which could be avoided using an objective assessment of physical activity such as accelerometer-based data in future studies. Sixth, we could not evaluate the impact of a uniform approach to statistical adjustment, because the degree of adjustment varied across studies. However, except for one study, all other studies adjusted for some established risk factors. Furthermore, there was no significant evidence of effect modification by the degree of adjustment on the association. Seventh, most of the studies were judged to be at serious risk of bias in at least one domain of the Cochrane risk of bias tool, but not at critical risk of bias in any domain. Finally, all physical activity exposures in each study were based on baseline assessments; hence, the potential for regression dilution bias, which could have potentially underestimated the associations. We propose large-scale studies with objective measures of physical activity and their repeat measures to better quantify the nature and magnitude of the prospective relationship between physical activity and pneumonia.

## Conclusion

Moderate-to-low quality evidence based on aggregate analysis of 10 prospective cohort studies demonstrates a lowered risk of incident pneumonia and mortality due pneumonia in general population participants who engage in regular physical activity.

## Supplementary Information

Below is the link to the electronic supplementary material.

Supplementary file1 (DOCX 339 KB)
