# Supplementary material for: Physical activity reduces the risk of pneumonia: systematic review and meta-analysis of 10 prospective studies involving 1,044,492 participants
Source: GeroScience. 2021 Nov 25;44(1):519–32. doi: 10.1007/s11357-021-00491-2 (PMC8811019; doi:10.1007/s11357-021-00491-2)
Supplement: Supplementary file 1 — Supplementary file1 (DOCX 339 KB) [file 11357_2021_491_MOESM1_ESM.docx]

**Supplementary Material**

| **Electronic Supplementary Material 1** | PRISMA checklist |
| --- | --- |
| **Electronic Supplementary Material 2** | MOOSE checklist |
| **Electronic Supplementary Material 3** | Literature search strategy |
| **Electronic Supplementary Material 4** | Reasons for exclusions during full text evaluation |
| **Electronic Supplementary Material 5** | Detail description of physical activity exposures by individual studies |
| **Electronic Supplementary Material 6** | Risk of bias assessment |
| **Electronic Supplementary Material 7** | Relative risks on exclusion of a study one at a time |
| **Electronic Supplementary Material 8** | Physical activity and risk of pneumonia in studies reporting age and/or sex adjusted estimates |
| **Electronic Supplementary Material 9** | Assessment of small study effects by funnel plot and Egger’s regression symmetry test |
| **Electronic Supplementary Material 10** | GRADE summary of findings |

**Electronic Supplementary Material 1.** PRISMA checklist

| **Section/topic** | **Item No** | **Checklist item** | **Reported on page No** |
| --- | --- | --- | --- |
| **Title** | | | |
| Title | 1 | Identify the report as a systematic review, meta-analysis, or both | 1 |
| **Abstract** | | | |
| Structured summary | 2 | Provide a structured summary including, as applicable, background, objectives, data sources, study eligibility criteria, participants, interventions, study appraisal and synthesis methods, results, limitations, conclusions and implications of key findings, systematic review registration number | 2 |
| **Introduction** | | | |
| Rationale | 3 | Describe the rationale for the review in the context of what is already known | Introduction |
| Objectives | 4 | Provide an explicit statement of questions being addressed with reference to participants, interventions, comparisons, outcomes, and study design (PICOS) | Introduction |
| **Methods** | | | |
| Protocol and registration | 5 | Indicate if a review protocol exists, if and where it can be accessed (such as web address), and, if available, provide registration information including registration number | Methods |
| Eligibility criteria | 6 | Specify study characteristics (such as PICOS, length of follow-up) and report characteristics (such as years considered, language, publication status) used as criteria for eligibility, giving rationale | Methods |
| Information sources | 7 | Describe all information sources (such as databases with dates of coverage, contact with study authors to identify additional studies) in the search and date last searched | Methods |
| Search | 8 | Present full electronic search strategy for at least one database, including any limits used, such that it could be repeated | Electronic Supplementary Material 3 |
| Study selection | 9 | State the process for selecting studies (that is, screening, eligibility, included in systematic review, and, if applicable, included in the meta-analysis) | Methods |
| Data collection process | 10 | Describe method of data extraction from reports (such as piloted forms, independently, in duplicate) and any processes for obtaining and confirming data from investigators | Methods |
| Data items | 11 | List and define all variables for which data were sought (such as PICOS, funding sources) and any assumptions and simplifications made | Methods |
| Risk of bias in individual studies | 12 | Describe methods used for assessing risk of bias of individual studies (including specification of whether this was done at the study or outcome level), and how this information is to be used in any data synthesis | Methods |
| Summary measures | 13 | State the principal summary measures (such as risk ratio, difference in means). | Methods |
| Synthesis of results | 14 | Describe the methods of handling data and combining results of studies, if done, including measures of consistency (such as I^2^ statistic) for each meta-analysis | Methods |
| Risk of bias across studies | 15 | Specify any assessment of risk of bias that may affect the cumulative evidence (such as publication bias, selective reporting within studies) | Methods |
| Additional analyses | 16 | Describe methods of additional analyses (such as sensitivity or subgroup analyses, meta-regression), if done, indicating which were pre-specified | Methods |
| **Results** | | | |
| Study selection | 17 | Give numbers of studies screened, assessed for eligibility, and included in the review, with reasons for exclusions at each stage, ideally with a flow diagram | Results and Figure 1 |
| Study characteristics | 18 | For each study, present characteristics for which data were extracted (such as study size, PICOS, follow-up period) and provide the citations | Results, Table 1 |
| Risk of bias within studies | 19 | Present data on risk of bias of each study and, if available, any outcome-level assessment (see item 12). | Results, Table 1 |
| Results of individual studies | 20 | For all outcomes considered (benefits or harms), present for each study (a) simple summary data for each intervention group and (b) effect estimates and confidence intervals, ideally with a forest plot | Results, Figure 2 |
| Synthesis of results | 21 | Present results of each meta-analysis done, including confidence intervals and measures of consistency | Results, Figure 2 |
| Risk of bias across studies | 22 | Present results of any assessment of risk of bias across studies (see item 15) | Table 1, Figure 3 |
| Additional analysis | 23 | Give results of additional analyses, if done (such as sensitivity or subgroup analyses, meta-regression) (see item 16) | Results; Figure 3; Electronic Supplementary Materials 7-8 |
| **Discussion** | | | |
| Summary of evidence | 24 | Summarise the main findings including the strength of evidence for each main outcome; consider their relevance to key groups (such as health care providers, users, and policy makers) | Discussion |
| Limitations | 25 | Discuss limitations at study and outcome level (such as risk of bias), and at review level (such as incomplete retrieval of identified research, reporting bias) | Discussion |
| Conclusions | 26 | Provide a general interpretation of the results in the context of other evidence, and implications for future research | Discussion |
| **Funding** | | | |
| Funding | 27 | Describe sources of funding for the systematic review and other support (such as supply of data) and role of funders for the systematic review | Funding section |

**Electronic Supplementary Material 2.** MOOSE checklist

**Physical activity reduces the risk of pneumonia: systematic review and meta-analysis of 10 prospective studies involving 1,044,492 participants**

| **Criteria** | | **Brief description of how the criteria were handled in the review** |
| --- | --- | --- |
| **Reporting of background** | |  |
| √ | Problem definition | Evidence on the association between physical activity and pneumonia is inconsistent |
| √ | Hypothesis statement | Regular physical activity is associated with reduced risk of pneumonia |
| √ | Description of study outcomes | Pneumonia |
| √ | Type of exposure | Physical activity |
| √ | Type of study designs used | Observational cohort studies |
| √ | Study population | Adult general populations with assessment of physical activity at study entry with at least 1 year follow-up |
| **Reporting of search strategy should include** | |  |
| √ | Qualifications of searchers | Setor K. Kunutsor, PhD; Samuel Seidu, MD |
| √ | Search strategy, including time period included in the synthesis and keywords | Time period: from inception to 15 September 2021  The detailed search strategy can be found in Appendix 3 |
| √ | Databases and registries searched | MEDLINE, Embase, Web of Science |
| √ | Search software used, name and version, including special features | OvidSP was used to search Embase and MEDLINE  EndNote X9 used to manage references |
| √ | Use of hand searching | We searched bibliographies of retrieved papers |
| √ | List of citations located and those excluded, including justifications | Details of the literature search process are outlined in the flow chart. The citation list for excluded studies is available on request. |
| √ | Method of addressing articles published in languages other than English | Not applicable |
| √ | Method of handling abstracts and unpublished studies | Excluded |
| √ | Description of any contact with authors | None |
| **Reporting of methods should include** | |  |
| √ | Description of relevance or appropriateness of studies assembled for assessing the hypothesis to be tested | Detailed inclusion and exclusion criteria are described in the Methods section. |
| √ | Rationale for the selection and coding of data | Data extracted from each of the studies were relevant to the population characteristics, study design, exposure, and outcome. |
| √ | Assessment of confounding | We assessed confounding by ranking individual studies on the basis of different adjustment levels and performed sub-group analyses to evaluate differences in the overall estimates according to levels of adjustment. |
| √ | Assessment of study quality, including blinding of quality assessors; stratification or regression on possible predictors of study results | The Cochrane Risk of Bias in Non-randomised Studies – of Interventions (ROBINS-I) tool was used to assess the risk of bias within individual observational studies |
| √ | Assessment of heterogeneity | Heterogeneity of the studies was quantified with I^2^ statistic that provides the relative amount of variance of the summary effect due to the between-study heterogeneity and explored using meta-regression and stratified analyses |
| √ | Description of statistical methods in sufficient detail to be replicated | Description of methods of meta-analyses, sensitivity analyses, meta-regression and assessment of publication bias are detailed in the methods. We performed fixed effects meta-analysis with Stata 17. |
| √ | Provision of appropriate tables and graphics | Table 1; Figures 1-3; ESM 6-9 |
| **Reporting of results should include** | |  |
| √ | Graph summarizing individual study estimates and overall estimate | Figure 2 |
| √ | Table giving descriptive information for each study included | Table 1 |
| √ | Results of sensitivity testing | Sensitivity analysis was conducted to assess the influence of omitting one study at a time on the pooled estimate. |
| √ | Indication of statistical uncertainty of findings | 95% confidence intervals were presented with all summary estimates, I^2^ values and results of sensitivity analyses |
| **Reporting of discussion should include** | |  |
| √ | Quantitative assessment of bias | Sensitivity analyses indicate heterogeneity in strengths of the association due to most common biases in observational studies. The systematic review is limited in scope, as it involves published data. Individual participant data is needed. Limitations have been discussed. |
| √ | Justification for exclusion | All studies were excluded based on the pre-defined inclusion criteria in methods section. |
| √ | Assessment of quality of included studies | Brief discussion included in ‘Methods’ section |
| **Reporting of conclusions should include** | |  |
| √ | Consideration of alternative explanations for observed results | Discussion |
| √ | Generalization of the conclusions | Discussed in the context of the results. |
| √ | Guidelines for future research | We recommend individual participant data meta-analysis |
| √ | Disclosure of funding source | In “Acknowledgement” section |

**Electronic Supplementary Material 3.** Literature search strategy

Relevant studies, published from inception to 15 September 2021 (date last searched), were identified through electronic searches without language restrictions using MEDLINE, Embase, and Web of Science databases. Electronic searches were supplemented by scanning reference lists of articles identified for all relevant studies (including review articles) and by hand searching of relevant journals.

| Database: Ovid MEDLINE(R) <1946 to present>  Search Strategy:  --------------------------------------------------------------------------------  1 physical activity.mp. or exp Exercise/ (288133)  2 exp Pneumonia/ (199050)  3 lower respiratory tract infection.mp. (3227)  4 cohort studies/ or longitudinal studies/ or follow-up studies/ or prospective studies/ or retrospective studies/ or cohort.ti,ab. or longitudinal.ti,ab. or prospective.ti,ab. or retrospective.ti,ab. (2865181)  5 2 or 3 (201668)  6 1 and 4 and 5 (235)  7 limit 6 to humans (228)  ***************************  Each part was specifically translated for searching the other databases (Embase and Web of Science) |
| --- |

**Electronic Supplementary Material 4.** Reasons for exclusions during full text evaluation

| **No.** | **PMID** | **Author_year** | **Data sources** | **Reason for exclusion** | **Further details** |
| --- | --- | --- | --- | --- | --- |
| 1 | 27780487 | Hemila, 2016 | Databases | Exposure not relevant | Assessed effect of vitamin E on pneumonia |
| 2 | 27757026 | Hemila, 2016 | Databases | Exposure not relevant | Assessed effect of vitamin E on pneumonia |
| 3 | 21386974 | Hemila, 2011 | Databases | Exposure not relevant | Assessed effect of vitamin E on pneumonia |
| 4 | 14747686 | Merchant, 2004 | Databases | Exposure not relevant | Assessed effect of antioxidants and vitamins on pneumonia |
| 5 | 30249944 | Ukawa, 2019 | Databases | Duplicate study | Duplicate study of Inoue, 2007 |
| 6 | 23041365 | Wojkowska-Mach, 2013 | Databases | Exposure not relevant | Physical activity exposure not relevant for this review |
| 7 | 33877614 | Chastin, 2021 | Databases | Review | Was a systematic review |
| 8 | 17398227 | Neuman, 2007 | Databases | Exposure not relevant | Assessed effect of vitamins on pneumonia |
| 9 | NA | Williams, 2014 | Reference list | Population not relevant | Elite runners and walkers |

**Electronic Supplementary Material 5.** Detail description of physical activity exposures by individual studies

| **Author, year of publication** | **PA exposure, type** | **PA ascertainment** | **Categorisation of PA** | **Risk comparisons used in analysis** |
| --- | --- | --- | --- | --- |
| Hamer, 2019 | Total PA, Aerobic plus resistance | Self-administered questionnaire administered at baseline. Ascertained frequency (number of days in the last four weeks) and duration (of an average episode) of participation in: domestic PA; light-intensity (slow/average pace) and moderate-intensity (fairly brisk/fast pace) walking; and type-specific sports and exercises | Categorised as inactive (not reporting any MVPA), insufficiently  active (> 0 < 150 min/week MVPA), or sufficiently active (at least 150 min/week MVPA). | Sufficiently active vs inactive |
| Inoue, 2007 | Playing sports, Aerobic plus resistance | Self-administered questionnaire administered at baseline | Categorised as <1, 1-2, 3-4, or >4 hours a week | >4 vs 1-2 hrs/week |
| Neuman, 2010 | Total PA, Aerobic plus resistance | Self-administered questionnaire administered at baseline and updated every two years. Assessed average time spent per week at various types of PA, such as walking, jogging, bicycling, and swimming, and also time spent performing leisure activity such as watching TV, driving, and sitting at home. Metabolic equivalents per hour score for recreational or leisure-time PA was calculated | PA was expressed in MET-hr/wk and categorised into quintiles | Top vs bottom quintile |
| Baik, 2000 | Total PA, Aerobic plus resistance | Self-administered standardised PA questionnaire administered at baseline | PA was expressed in MET-hr and categorised into quintiles | Top vs bottom quintile |
| Paulsen, 2017 | Total PA, Aerobic plus resistance | Self-administered questionnaire was used to ascertain leisure time weekly amount of light (not sweaty or breathless) and vigorous (sweaty or breathless) PA during the past year | PA was categorised as none (no vigorous or light activity), slightly (< 3 h of weekly light  activity and no vigorous activity), moderately (≥3h of weekly light activity or < 1 h of vigorous activity) or highly (≥1 h of vigorous activity per week) physically active | High vs none |
| Hemila, 2006 | Leisure PA, Aerobic plus resistance | Self-administered questionnaire was used to ascertain the intensity of average PA during leisure time over the previous 12 months with the following alternatives: 1) light: reading, watching TV, listening radio, or going to movies, mostly activities that are not physically loading; 2) moderate: walking, fishing, hunting, or gardening quite regularly; and 3) heavy: actual physical exercises, such as jogging, skiing, swimming, gymnastics, court and field sports quite regularly | Categorised as light, moderate and heavy | Heavy vs light |
| Ogunmoroti, 2016 | Total PA, Aerobic plus resistance | PA was assessed using a detailed questionnaire which identifies the time and frequency spent in activities during a  typical week in the previous month using 28 questions including household chores, lawn/yard/garden/farm, care of children/adults, transportation, walking (not at work), dancing and sport activities, conditioning activities, leisure activities, and occupational and volunteer activities. Minutes of walking,  conditioning, and leisure activities were also included as exercise, and the minutes of moderate and vigorous exercise  were calculated from the questionnaire | A point scoring system was used where points are assigned and summed: ideal=2 points, intermediate=1 point, and poor=0 point, for a total score ranging from 0 to 14 points. Study  participants who scored 0 to 8 points were classified as inadequate, those who scored 9 or 10 points were classified as average, and participants who scored 11 to 14 points were  classified as optimal. PA was categorised as ideal, intermediate and poor | Ideal vs poor |
| Ahmadi, 2021 | Total PA, Aerobic plus resistance | PA was measured using a self-administered questionnaire which included items on frequency and duration of walking, moderate intensity activity,  and vigorous intensity activity. PA was expressed as MET min/week. Participants who attained 600 MET-min/week met the PA guidelines of 150 min of moderate-vigorous physical activity a week | Participants were classified as inactive if they 0 MET-min/  week, insufficiently active if they had*<*600 MET-min/week, and sufficiently  active if they had at least 600 MET-min/week. | Sufficient vs inactive |
| Ikeda, 2020 | Walking, Aerobic | Self-administered questionnaire was used to ascertain daily walking habits based  on responses of the question ‘In your daily life, do you walk or do any equivalent amount of physical activity for  >1 h a day?’ with possible answers of ‘yes’ or ‘no’ | PA was categorised into walking vs no walking habits | Walking vs no walking habits |
| Jackson, 2016 | Exercise, Aerobic | Self-administered questionnaire administered at baseline and updated every two years. Used to assess weekly exercise such as aerobics, biking, swimming, walking and weight training | Weekly exercise was categorised into 0, 1-3, and 4-7 days | 4-7 vs 0 days/week |

MVPA, moderate- and vigorous-intensity physical activities; PA, physical activity

**Electronic Supplementary Material 6.** Risk of bias assessment

**Electronic Supplementary Material 7.** Relative risks on exclusion of a study one at a time

**Electronic Supplementary Material 8.** Physical activity and risk of pneumonia in studies reporting age and/or sex adjusted estimates

**Electronic Supplementary Material 9.** Assessment of small study effects by funnel plot and Egger’s regression symmetry test

**Electronic Supplementary Material 10.** GRADE summary of findings

**Question**: [Most physically active] compared to [Least physically active] for [Pneumonia]

**Setting**: General population

| **Certainty assessment** | | | | | | | **Effect** | | **Certainty** |
| --- | --- | --- | --- | --- | --- | --- | --- | --- | --- |
| **№ of studies** | **Study design** | **Risk of bias** | **Inconsistency** | **Indirectness** | **Imprecision** | **Other considerations** | **Relative (95% CI)** | **Absolute (95% CI)** |  |
| Pneumonia incidence or death in overall population | | | | | | | | | |
| 10 | observational studies | serious ^a^ | not serious | not serious | not serious | Magnitude of effect^d^ Plausible residual confounding^f^  dose response gradient^g^ | **RR 0.69** (0.64 to 0.74) | **1 fewer per 1,000** (from 1 fewer to 1 fewer) | ⨁⨁⨁◯ Moderate |
| Pneumonia incidence | | | | | | | | | |
| 6 | observational studies | serious ^b^ | not serious | not serious | not serious | Magnitude of effect^e^ Plausible residual confounding^f^  dose response gradient^g^ | **RR 0.82** (0.72 to 0.93) | **1 fewer per 1,000** (from 1 fewer to 1 fewer) | ⨁⨁◯◯ Low |
| Pneumonia-related death | | | | | | | | | |
| 4 | observational studies | serious ^c^ | not serious | not serious | not serious | Magnitude of effect^d^ Plausible residual confounding^f^  dose response gradient^g^ | **RR 0.64** (0.59 to 0.70) | **1 fewer per 1,000** (from 1 fewer to 1 fewer) | ⨁⨁⨁◯ Moderate |

**CI:** Confidence interval; **RR:** Risk ratio

**Explanations**

a. The overall risk of bias in 9 out of 10 studies was serious risk of bias.

b. The overall risk of bias was serious.

c. The overall risk of bias was serious.

d. Strong association

e. Association not strong

f. There is no evidence that the influence of all plausible confounding would reduce a demonstrated effect or suggest a spurious effect when results show no effect

g. Evidence of a dose-response gradient
